# Nudging in the time of coronavirus? Comparing public support for soft and hard preventive measures, highlighting the role of risk perception and experience

**DOI:** 10.1371/journal.pone.0256241

**Published:** 2021-08-13

**Authors:** Levente Dudás, Richárd Szántó

**Affiliations:** Department of Decision Sciences, Corvinus University of Budapest, Budapest, Hungary; Middlesex University, UNITED KINGDOM

## Abstract

The importance of researching public support for preventive policies have been amplified by the COVID-19 pandemic. Using a representative sample of the Hungarian population, we investigated the support for commonly used preventive measures (social distancing, hand hygiene and wearing masks) comparing two different policy tools (nudges and regulations). Because of the high risk and unfamiliarity of the pandemic, the respondents’ risk perception and experience with the disease was also assessed. All preventive measures were generally supported and, contrary to the findings of previous nudge research, there was no clear pattern whether regulations or nudges are preferred. People with higher level of risk perception supported both types of policies more but slightly favoured the regulations. Those who had contact with the disease (either themselves or a close friend or family member contracting COVID-19) reported a higher level of risk perception. When the person themselves was afflicted, this higher levels of risk perception did not translate to a higher level of support, moreover, it even decreased support for the regulations according to regression analysis. In case of a loved one contracting the disease, there was an increased support for both types of measures, but that is explained by the higher risk perception.

## 1 Introduction

Since the seminal book of Thaler and Sunstein [1] policymakers have started to actively and consciously apply nudges. Nudges are defined as ‘… any aspect of the choice architecture that alters people’s behavior in a predictable way without forbidding any options or significantly changing their economic incentives.’ [1, p. 6]. Numerous studies have investigated the effectiveness of nudges across a variety of domains, as summarized in the study of Szászi et al. [2], and it was specifically demonstrated that nudges can be effective in promoting healthy behavior [3], environmentally conscious efforts [4], encouraging retirement savings [5], increasing customer satisfaction and firm’s profitability [6], and in many other fields. The global pandemic presented a situation, however, in which the application of the libertarian-paternalistic approach needs to be re-evaluated.

Effectiveness of a behaviorally informed policy is not a guarantee to apply with success. The success of interventions concerning for example the combat against pandemics such as SARS, H1N1 or COVID-19 also depends heavily on the level of public support [7], because that support corresponds directly to the intent to comply with the policies [8]. Public acceptance of nudges is a well-researched topic [9], and most studies revealed that the majority of people accept these types of interventions [see 10, for a review]. Several studies demonstrated that people prefer the softer approaches over restrictive measures or taxation [11, 12], and nudging has grown popular in recent years among policymakers, because of this relative popularity. Another appeal of nudges is that the less intrusive approach makes them applicable where harsher measures are not tenable. However, the behaviorally informed policies are not only complementing the traditional tools, they also compete with them in a sense that policymakers can use these different approaches as alternatives [13].

The considerations about competing ‘hard’ and ‘soft’ approaches have received increasing attention by researchers and practitioners. Bans and mandates receive less support than behavioral interventions even when the outcomes of strong regulatory policies are more desirable to the people [14]. Introducing these softer approaches can even be counterproductive. A study shows that support for carbon taxes drop when a green nudge is also introduced, and so a nudge may crowd out support for more effective, but more burdensome measures, like taxes [15]. Still, Benartzi et al. [16] suggest that the cost to impact ratio of nudges is better than their policy alternatives.

With more and more governments adding the libertarian-paternalistic approach into their policy toolbox [17] interest among researchers has increased to understand the underlying reasons for the support or rejection of nudges. One aspect of this understanding is how the quality of policies and the people themselves contribute to these decisions. Jung and Mellers [18] had helped to understand a great deal about how individual dispositions, and perceptions of nudges contribute to the level of support for them, while Reynolds et al. [19] showed that support depends heavily on perceived effectiveness as well as perceived fairness, personal beliefs, values of the individual, and demographic variables.

The importance of these topics in nudge research (the soft versus the hard approach, individual attitudes and perceptions) are further amplified by the highly pressing issue of the global pandemic that defined 2020. COVID-19 has also brought forth new challenges that needed answering from policymakers. What kind of preventive measures to implement has become an urgent question. Issuing strict restrictive regulations or suggesting preventive behavior only through indicative nudges were competing alternatives.

In this paper we take a closer look at public support for these options using a survey instrument carried out when COVID-19 cases hit critical levels for the first time in the country, with respondents representative of the Hungarian population, and investigate three aspects that the pandemic gave new meaning to. Firstly, to what extent are the restrictive actions, regulations supported compared to the softer approach of behaviorally informed policies. Secondly, we examine how the perceived risk of the virus is influencing the support for the preventive measures, and whether it influences the preference between the nudges and regulations. Lastly, we take a look at how first-hand experience with the disease connects to risk perception and support for these policies.

## 2 Background

### 2.1 Types of nudges

The great appeal of nudges is that they preserve autonomy while significantly influencing behavior [20]. These characteristics of nudges can even be more welcomed when individual liberties are otherwise curbed. Previous research showed, however, that support for specific nudges can fall on a wide range, therefore we are reflective on the types of nudges we include in our study.

We can distinguish nudges based on the cognitive mechanism by which the nudges operate; system 1 & system 2 [21], which also relates to the transparency of this mechanism; overt and covert [22]. System 1 type nudges mostly influence automatic, heuristic decision-making processes, while system 2 nudges enable deliberative processing, and for this reason, they are generally seen as more acceptable [18]. In the context of a pandemic, we have seen that the system 2 type nudging, which consists of educational and cautionary messaging, is usually applied together with other preventive measures to complement them. Therefore, we included system 1 type nudges into our study.

According to Hagman et al. [23] there is a difference in acceptability of nudges regarding the beneficiary of the intervention. Pro-social nudges, which steer people toward socially beneficial behavior, even if it conflicts with maximizing private welfare, are less acceptable compared to pro-self nudges. In this regard, preventive nudges against the virus are all similar and, while they are both protecting the actor as well as their environment, they can be seen as pro-social acts. There is mounting evidence that nudges receive broad support everywhere in the world, especially in the domain of health [10, 24, 25], but the level of acceptance of nudges does vary across cultures [17], and even subcultures [26]. In some countries (like in China or South Korea) they are accepted with enthusiasm [10], while in most western democracies people tend to provide them green light with some modest reservations, and there are countries (like Denmark or Hungary) where citizens are more cautious, and reluctant to support behaviorally informed policies [27]. This latter is relevant for our study as it was conducted with Hungarian subjects.

### 2.2 Preventive measures against COVID-19

Behavioral change is a crucial factor to contain the spread of the COVID-19 disease. Previous findings of social science can help the creation of preventive policies, as it was outlined in the summary of Bavel et al. [28]. Policy makers should capitalize on the results of research about risk perception, science communication, aligning individual and collective interests amongst many other areas. Capraro et al. [29] also provide insights on how to boost cooperation, which is essential during a pandemic.

Many of the large number of studies, that were published about the COVID-19 disease, concern the use of nudges and the majority of them endorse their application. Weijers et al. [30] reported about a successful field experiment: with different types of nudges (increasing attention and emphasizing the gains of hand sanitation) they were able to boost disinfectant use in Dutch shops. In another study, the authors report that local Chinese governments were able to increase the positive effect of two way risk communication on people’s willingness to comply with COVID-19 policies by utilizing nudge interventions like descriptive social norms and infotainments [31]. In a Japanese study nudged-based messages were sent to mobile devices to convince people to avoid crowded places and close contacts [32]. Through location tracking, mobility of the recipients was monitored and, under some conditions, these nudges were proved to be successful and cost effective. Prasetyo and Sofyan [33] tested five visual campaigns to reduce travel intentions during the Eid festive season in Indonesia, and they found some of them effective in making people reconsider their plans to travel. Debnath and Bardhan [34] consider the concept of nudging very broadly, and so they identify a great amount of nudges that were applied in 14 different policy sectors in India, which were concluded to have a measurable positive effect.

Some studies, however, have found mixed, or no results from the application of these behaviorally informed policies. An experimental research used social norms to steer people’s behavior toward compliance with social distancing and lockdown measures, but they had mixed results about the effectiveness of this intervention [35]. Blackman and Hoffmann [36] did not find evidence that informational (system 2) nudges would boost intended compliance with COVID-19 regulations studying the attitudes of Colombian young adults, yet these nudges raised their level of concern about the disease. The right framing of cautionary messages is also a mechanism that can steer people toward compliance with preventive policies, but their effective application may be context dependent. In two studies with US participants Banker and Park [37] found in the initial weeks of the COVID-19 outbreak that messages in social media framed ‘protect yourself’ or ‘your loved ones’ generated more interest than a pro-social frame (‘protect your community’), while Capraro and Barcelo [38] found some weeks later that the frame ‘protect your community’ increased the intention to wear a face mask the most.

It seems essential for an effective pandemic defense, that the nudges are complimented with other approaches, and beyond these campaigns governments introduce “hard” rules as well. The use of solely nudge theory by the UK government at the early stage of the pandemic received strong criticism in March of 2020 due to the lack of drastic measures [39]. The way to attain the necessary behavior changes can not only be achieved by nudges of course, but there are several other instruments like incentives, communication, bans, and mandates [40]. In our research we compare the application of nudges with regulatory measures as these were by far the most used policies to combat the spread of the disease. When formulating the survey, we applied these policy interventions (regulations and system 1 type nudges) on the most frequently used infection control areas, namely: hand hygiene, social distancing, and mask wearing.

Although we agree with research cited in the Introduction that nudges are generally more well-received by the people than some stronger policy interventions, our study questions the universality of this claim. We argue that in some contexts stricter policies are more welcomed by the public. If people do not believe that certain soft policy measures can attain the necessary societal effect, they may prefer stronger regulations over nudges. Especially in high stakes situations where the effectiveness of policy interventions may result in fewer fatalities or lower incidence rates of a serious disease.

### 2.3 Risk perception and policy support

There is a wide consensus in the literature that risk perception influences policy support across various domains. Drews and Berg [41] claim for example that risk perception positively influences public support for climate policies; people who believe in immediate and severe negative consequences of climate change, respond to the policies in a reassuring way. Zahran et al. [42] also found a robust effect of subjective risk perception on climate policy support, while objective risk measures (like living close to coastal areas) explained only little variance of public support. In a totally different domain similar patterns can be observed: a perceived high risk of terrorism predicts preferences towards massive governmental spendings on counter-terrorism [43]. Nevertheless, our knowledge on the risk perception–policy support relationship is still fairly limited, as Gerber et al. [44, p. 397] point out: ‘relatively less attention has been devoted to explaining whether perceived risk systematically shapes an individual’s views of public policies designed to manage possible hazards.’

The influence of risk perception on public acceptance of nudges in particular has been tested only in a few studies so far. Sunstein et al. [17] did not find any significant relationship between risk perception and overall nudge approval. Risk perception in this study, however, was measured with one very general question. Bates et al. [45] found positive relationship between the awareness of the link between alcohol consumption and cancer (i.e., awareness of the risk) and the public support of alcohol policies. The study did not investigate the approval of nudges specifically, but these types of interventions were included into the set of policies they looked at.

The unprecedented elevated risk, which the global pandemic has brought, emphasizes the re-evaluation of this psychological attribute and its relation to policy support. We can intuitively expect that higher risk perception corresponds with the backing of preventive measures. In our study we applied a set of COVID-19 related questions developed by Dryhurst et al. [46] to assess the risk perception of our respondents and relate that to policy support.

### 2.4 Personal experience with COVID-19

Numerous studies have confirmed that personal experience is a principal predictor of risk perception. For example, personal experience with extreme weather events (heat waves, flood, etc.) increases the perceived risk of climate change [47], women focused more on the risk of breast cancer after a diagnosis of a family member or close friend [48], and direct exposure to the coronavirus increases the perceived risk of COVID-19 [46]. Nonetheless, the role of direct experience on public acceptance of behavioral interventions has been rarely investigated. Sunstein et al. [17] found that drinkers did not support nudging in general, while smokers found government campaigns against smoking unacceptable. These direct activity-based rejections may not be expected in this current study, since the risks associated with the contraction of COVID-19 were surrounded with a great deal of uncertainty, and it affects not just the person but others as well.

Based on the relationship between first-hand experience and risk perception, we expect that first-hand experience with COVID-19 will elicit a greater level of approval of both regulatory and nudge policies. Those who experienced the consequences of the disease directly (because they were affected) or indirectly (through a family member or a close friend) may have higher risk perception and greater support for the preventive measures.

### 2.5 COVID-19 situation at the time and place of data collection

The survey was conducted in Hungary between the 16^th^ and 20^th^ November 2020, just after stricter regulations were put into place in the country because of daily new COVID-19 cases had increased to a critical level for the first time. We represent the strictness of containment measures based on Hale et al. [49] represented over time by the line on S1 Fig, along with the number of registered new cases over time represented by bars. The course of the first and second waves of the COVID-19 pandemic in Hungary was similar to other Central European countries, with some minor variations. In the first wave Hungary saw a small number of cases and fatalities compared to Western European countries, but the restrictions were nonetheless in place. In the second wave, starting 2020 autumn, incident rates and fatalities were higher. Up until the time of data collection regarding hand hygiene and social distancing there were no mandates decreed, only nudges were applied, but policies for mask wearing went from strict to stricter, after mandating their use in public places indoors since the end of April, their use in public urban settings outdoors were also required starting mid-November.

## 3 Materials and methods

### 3.1 Participants and procedure

One thousand Hungarian participants were recruited for this study. The demographic characteristics of the sample, in terms of our criteria for representative sampling, is presented in Table 1. An online survey design was applied, and the questionnaire was administered as part of a CAWI (Computer Assisted Web Interviewing) omnibus survey. The sample was representative of the adult Hungarian population under the age of 64 in terms of demographics (age, gender, education), with a slight possible bias of computer proficiency due to the CAWI nature of our survey. As is normal practice for qualitative sociological research, every effort has been made to preserve the anonymity of all interview participants. All participants provided informed consent in a written format. The research has received the relevant ethics approval form the ‘CUB Research Ethics Committee’ of the university the authors are affiliated to.

**Table 1:** Composition of the sample.

		% Sample
Age	18–24	12%
	25–34	20%
	35–44	25%
	45–54	22%
	55–64	21%
Sex	Female	50%
	Male	50%
Education	Primary	24%
	Secondary	56%
	Tertiary	20%

### 3.2 Measures

Risk perception was measured with a COVID-19 risk perception index that covers affective, cognitive, and temporal-spatial dimensions of risk perception based on Dryhurst et al. [46]. The construct contains six items: (1) level of worry, (2) perceived likelihood of direct personal effects, (3) perceived likelihood of direct effects on family members and friends, (4) personal beliefs about how many people in the country will be affected, (5) perceived probability of getting sick, and (6) getting sick seriously. In case of each item a 7-point scale was used (see Table 2 items for exact items and scales). The index was calculated as a simple arithmetic mean of the individual items (*α* = 0.826).

**Table 2:** Survey items for risk perception index [based on: 46].

Construct item	Scale
How worried are you personally about the coronavirus epidemic?	Seven point Likert scale, 1 = not at all worried, 7 = very worried
What do you think about the following statement? The coronavirus will NOT affect very many people in Hungary.	Reverse coded, Seven point Likert scale, 7 = strongly disagree, 1 = strongly agree
What do you think about the following statement? Getting sick with the coronavirus can be serious.	Seven point Likert scale, 1 = strongly disagree, 7 = strongly agree
How likely do you think it is that your family members or close friends in Hungary will catch the coronavirus in the next 6 months?	Seven point Likert scale, 1 = not at all likely, 7 = very likely
How likely do you think it is that you will catch the coronavirus in the next 6 months (if you had it already, you will catch for the second time)?	Seven point Likert scale, 1 = not at all likely, 7 = very likely
What do you think about the following statement? I will probably get sick with the coronavirus in the next 6 months.	Seven point Likert scale, 1 = strongly disagree, 7 = strongly agree

Table 3 presents three hard and three soft policy measures that participants had to rate, to what extent they supported them. Responses were given in a seven point scale ranging from 1 (certainly oppose) to 7 (certainly support). The policies we call softer, can be categorized as system 1 nudges, while the harder policies, are strict mandates also called regulations. For further analysis, focusing on the difference in attitude toward the two types of policies, we calculated a nudge support and a regulation support index as the average of corresponding survey items (Cronbach’s alpha for regulation support *α* = 0.859, nudge support *α* = 0.862), and also constructed a variable measuring the difference in support between regulations and nudges for each of the three prevention areas (*α* = 0.2). When these difference variables have positive values, the regulatory intervention was rated higher than the corresponding nudge policy.

**Table 3:** The wording of the 3 hard and 3 soft policy measures.

Preventive Measure	Regulation (hard approach)	Nudge (soft approach)
Hand hygiene	People are not allowed to enter public places without disinfecting hands at the entrance	At the entrance of public places painted footprints on the floor lead to sanitizers with colorful instructions
Social distancing	People are not allowed to enter public places until the number of people is below a limit when the 1.5-meter distance can be guaranteed	In public places stickers on the floor show 1.5-meter distance, to notify about the importance of social distancing
Wearing mask	People are not allowed to enter public places without wearing mask hiding their nose and mouth	At the entrance of public places posters highlight that 9 out of 10 people wear mask to combat with the pandemic

The respondents’ experience with COVID-19 was also assessed in the survey. For the purposes of this study, direct experience means the participant contracted or thought they contracted COVID-19, whereas indirect experience means a family member or close friend had contracted it. When testing direct experience respondents had to answer whether they had COVID-19 or not. We also included an option for stating that the respondent thought that he or she was infected with coronavirus but was not tested. Those who actually had a positive COVID-19 test and those who thought to have had the disease were coded as one group. Surveying indirect experience, the following question was presented to the participants: “To the best of your knowledge, was anyone in your direct environment (family members, close friends) infected with the coronavirus?” (dichotomous, yes or no scale).

### 3.3 Statistical analysis

For our first research question, how the support for nudges and regulations compare in an increasingly risky environment, we evaluate the responses for policy support in two different ways. From one angle, we are interested in the ratio of those who supported the policies (given answers on the positive side of the scale) compared to those who did not (neutral or negative). We test the association between this dichotomized support and the policy types for each prevention area separately using contingency table based *χ*^2^ tests. We also evaluate the support for policies looking at the original distribution of responses given on the seven point Likert scale. Normality of distribution is not confirmed by Shapiro-Wilk tests for the six policy support variables, regulations and nudges assessed on the three prevention areas, therefore we test the difference in support using nonparametric Wilcoxon–Mann–Whitney tests.

Next, we aim to investigate the association between risk perception and policy support, with particular focus on differences regarding the policy types. We are interested to see, if there is a shift in preference in terms of policy strictness among people with higher levels of risk perception. To test this, we calculate Pearson correlation coefficients between the risk perception index and the policy support measures employing the constructed indicies for risk perception, nudge support and regulation support, as well as the difference in support variables. The low Cronbach alpha value (0.2) for the difference in support indicates contrasting relations across the preventive areas we investigated, therefore we calculate the correlation of risk perception and the support variables for the three areas separately as well.

The last aspect of the research concerns the respondents’ experience with COVID-19, and how that affects the individuals risk perception, or preference in policy strictness. We defined the experience as it can occur directly (contracting the disease) or indirectly (a close friend or family contracting it). The occurrence of the two experiences can obviously be related and, consequently, examining their cross relation should not be neglected. We take the Cartesian product of the two binary, experience variables and evaluate the mean risk perception, support for regulations and support for nudges across the four groups using the constructed indicies. Since the aggregated indicies were also not found to be normally distributed based on Shapiro-Wilk tests, we use the nonparametric Kruskal–Wallis test for one-way analysis of variance to determine differences in distribution among the groups defined by experience, and complement it with pairwise analysis using Mann–Whitney tests with Bonferroni correction to correct for multiple comparisons when calculating significance. This provides us with an understanding of the association between the experience with the disease and the other main variables separately, but to be able to focus more intently on the interaction of experience and risk perception, and how they can correspond to support for the policies, we also fit a regression model. Nudge support index and the regulation support index are defined as dependent variables in the OLS regression models, and risk perception index, the experience variables (examining models including just one or both together), and the interaction terms between risk and experience are included as independent variables. Besides measuring the extent these variables can predict support for the policies, when we interpret the results, we will also be interested in the differences in model composition for the two types of policies.

## 4 Results and discussion

### 4.1 Preventive measures

Our results reveal, that public policy measures were generally supported, but there is no clear pattern to whether hard or soft policy measures are preferred across the preventive strategies we looked at. Our takeaway from this is that in situations when the associated risk is high, in contrast to more ordinary settings, hard policy measures receive the same or higher approval ratings than nudge applications. S2 Fig reflects what percentage of respondents supported the policies, and in Table 4 we also report the mean support for them. The differences in support for the two types of policies are analogous when we evaluate them using the means or percentages. In case of hand hygiene there is no significant difference between the two types of measures (*χ*^2^ = 1.187, p = 0.276). Respondents viewed nudge intervention more acceptable regarding social distancing (*χ*^2^ = 19.761, p < 0.001), while the regulation prescribing wearing mask was rated higher than nudging (*χ*^2^ = 53.35, p < 0.001). When interpreting these results, one should consider that wearing masks was an exceptionally hot topic in Hungary at the time of the data collection. The Hungarian government tightened the rules for mask wearing shortly beforehand. The recently introduced stronger policies might also have increased the approval ratings of mask wearing policies.

**Table 4:** Mean support for preventive measures.

	Regulations		Nudges	
	*Mean Support*	*St.Dev*.	*Mean Support*	*St.Dev*.
Hand hygiene	5.21	1.64	5.62	1.37
Social distancing	5.6	1.51	5.59	1.39
Wearing mask	5.9	1.52	5.37	1.59

Note. The table reports summary statistics of policy support assessed on a seven point Likert scale coded 1 through 7.

Some cross-country studies stated that in Hungary (along with Denmark and Japan) people are very reluctant to accept nudge policies [17]. Our results suggest a more nuanced view about countries that are not nudge enthusiasts according to these studies. All three nudges we investigated received fairly high approval ratings in the Hungarian context, meaning when people’s risk perception is high, they may approve nudges that reduce risk whatever their preferences regarding nudges in general. Hence, public support of behavioral intervention policies is domain specific and context dependent. This finding is supported by other studies too. Jung and Mellers [18] mention that despite the general public approval of nudges in the US, some interventions were still opposed. Meanwhile, mandatory subliminal advertising to discourage smoking and overeating was widely rejected in the study of Reisch and Sunstein [27] even in countries with generally high nudge support.

### 4.2 The influence of risk perception on policy support

The level of support for policies in our sample is demonstrated above, and before sharing results about its association with risk perception, we report descriptive statistics about the risk factors of coronavirus. As the means in Table 5 indicate, the perceived risk of our respondents was fairly high, with the virus affecting many people and the sickness being serious the most agreed upon components of this assessment.

**Table 5:** Descriptive statistics on risk perception.

	Mean	St.Dev.
Risk perception index (aggregate)	4.77	1.11
Worried about	4.65	1.63
Affect many	5.54	1.49
Sickness is serious	5.57	1.32
Likely in family	4.86	1.59
Likely to contract	4.13	1.56
Likely get sick	3.89	1.54

Note. The table reports summary statistics of risk perception about COVID-19 assessed on a seven point Likert scale coded 1 through 7.

We hypothesized that those with high risk perception would favor regulations and, among them especially, the advantage of the softer approach showed in other studies would diminish. Table 6 presents that there is a moderate correlation between the risk perception index and the approval of both regulatory and nudge interventions. This level of correlation is similar across all prevention areas. Moreover, there is a relatively weak, but significant positive correlation between the risk perception index and the difference variables, meaning that people with a higher level of risk perception favor regulatory approaches slightly even more. We mentioned that recent policy changes could have influenced responses about mask wearing, and given that the individual respondents’ difference in policy support correlated more strongly with risk perception in case of mask wearing, these changes may have also polarised the responses. We reason, that those who did not think the more strict regulations are necessary, or did not intend to comply, showed more support for nudges.

**Table 6:** Correlations between risk perception and policy support.

	Mean Support		Hand Hygiene		Social Distancing		Wearing Mask	
	r	p	r	p	r	p	r	p
Risk Perception—Support Regulation	0.55	<.001	0.46	<.001	0.45	<.001	0.55	<.001
Risk Perception—Support Nudge	0.50	<.001	0.45	<.001	0.46	<.001	0.42	<.001
Risk Perception—Difference in Support			0.07	.02	0.10	.0012	0.13	<.001

Note. The table reports correlations (Pearson’s r) and significance (using Bonferroni adjustment) between risk perception and the different preventive policies. The ‘mean support’ and ‘difference in support’ are calculated variables.

Based on our findings, risk factors considered to be exceptionally harmful and threatening by the public may push citizens towards the direction of accepting stronger regulations. This result explains—at least to a certain extent—why many people in British and Dutch societies were shocked and skeptical with the approach their government represented in the early days of the coronavirus crisis [39]. People’s risk perception index was one of the highest in the UK in the Spring of 2020 according to a comparative study [46], consequently they would have expected stronger measures. Solely using nudge interventions seemed insufficient to them when considering the risk they perceived. Earlier studies confirmed that bans, mandates, taxes, etc. were less popular than nudges [9, 11, 12], but our results suggest that when the perceived risk is high, governments should not rely exclusively on nudge interventions if they want to gain support and compliance from their citizens.

### 4.3 Experience with COVID-19

A very small number of our respondents (2.5%) tested positive for COVID-19 prior to the data collection, which was approximately equal to what official statistics reported at that time. However a bigger group, an additional 13.7% of the respondents were convinced that they had been infected with the virus, but they did not have themselves tested. This seems exaggerated, but somewhat believable due to the very high ratio of positive tests during the second wave in Hungary (climbing over 20% in 2020 November) suggesting a much higher infection rate than the official number. To evaluate the direct experience we coded both the tested and untested groups as ‘Yes’. About the indirect experience, 34.6% of our sample asserted that their family members and/or close friends were affected by the disease.

We tested how the combination of these experiences are associated with risk perception and policy support and report the results in Table 7. The Kurskal Wallis tests indicate that at least one of these groups stochastically dominates one other group regarding risk perception, (*χ*^2^ = 85.5, df = 3, p <.001) regulation support (*χ*^2^ = 13.1, df = 3, p = .004) and nudge support (*χ*^2^ = 11.2, df = 3, p = .011). The pairwise comparisons show significant differences in cases where the difference in mean ranks are larger: the ‘no experience’ group differs from all the others regarding risk perception, and the ‘indirect experience only’ group differs from the ‘direct experience only’ group in terms of policy support (both nudge and regulation). The latter suggests that direct experience may decrease, while indirect experience may increase the support for the policies.

**Table 7:** Cross relation of direct and indirect experience.

		Risk Perception		Support for Regulations		Support for Nudges	
Experience	N	Mean	Mean Rank	Mean	Mean Rank	Mean	Mean Rank
No experience	595	4.51	431	5.50	489	5.49	494
Indirect experience only	243	5.16	603	5.81	546	5.70	535
Direct experience only	59	4.98	566	5.16	412	5.10	401
Both direct and indirect	103	5.23	620	5.64	512	5.58	513

To investigate the role of experience further, and test to what degree are variations in policy support explained by experiences with COVID-19, risk perception and their effect on each other, we evaluated linear regression models and report the results in Table 8). In four simple models to explain the support for policies [M1—M4] we included the risk perception index (RPI) and either one of the experience variables as well as their interaction with the respondent’s RPI. The coefficient of the risk perception is strong and significant in all of these models.

**Table 8:** Regression models on policy support.

	Support Nudge [M1]	Support Regulation [M2]	Support Nudge [M3]	Support Regulation [M4]	Support Nudge [M5]	Support Regulation [M6]
Risk Perception	0.629 ***	0.761 ***	0.606 ***	0.707 ***	0.64 ***	0.767 ***
	(0.048)	(0.05)	(0.036)	(0.038)	(0.049)	(0.051)
Direct Experience	-0.826.	-1.229 *			-0.748	-1.176 *
	(0.498)	(0.515)			(0.512)	(0.53)
RP x DE	0.082	0.157			0.072	0.149
	(0.096)	(0.099)			(0.099)	(0.102)
Indirect Experience			-0.355	-0.373	-0.195	-0.138
			(0.372)	(0.386)	(0.38)	(0.393)
RP x IE			0.037	0.047	0.02	0.016
			(0.073)	(0.075)	(0.074)	(0.077)
Constant	2.372 ***	1.765 ***	2.602 ***	2.164 ***	2.317 ***	1.73 ***
	(0.249)	(0.258)	(0.186)	(0.193)	(0.256)	(0.265)
F statistic	120.21	154.48	113.96	145.76	72.4	92.65
	(3, 996)	(3, 996)	(3, 996)	(3, 996)	(5, 994)	(5, 994)
Prob > F	<.001	<.001	<.001	<.001	<.001	<.001
Adj. R 2	0.264	0.315	0.253	0.303	0.263	0.314

Note: The table reports the estimates of linear models where the outcome variable is the mean support for nudges or regulations. Standard errors are in brackets for the input variables. The categorical experience variables were specified to be a contrast centered at 0. They have the value 1 in case of an experience and -1 in absence of it. RP abbreviates the risk perception index, DE the direct experience, IE the indirect experience. The interaction terms are denoted using (x). Significance levels are denoted with (.) when p < 0.10; (*) when p < 0.05; (**) when p < 0.01; (***) when p < 0.001.

Models [M1] & [M2] show that the direct experience does have a negative effect on policy support, which is significant in case of regulations and, also marginally significant for nudges. In models [M3] & [M4] we see that indirect experience does not have a significant main effect alongside the risk perception for either policies. This suggests that any affect on policy support from the indirect experience is fully explained by the higher risk perception fueled by that experience and the social amplification of risk [50]. If we include both types of experiences in the models [M5] & [M6], we see that only the coefficient for the RPI is significant for the mean nudge support and, in case of regulations, direct experience also significantly contributes to support, decreasing it. The interaction effects are not significant in models [M1—M6], meaning that there is no evidence that the effect of risk perception on policy support is conditional on the subjects’ experience.

Somewhat surprisingly, there seem to be two contradicting effects on policy support for people who already contracted the disease. While the experience’s contribution to a higher risk perception should increase the level of support, there is also a tendency to support the preventive measures less. One should note that people who had COVID-19 are believed to develop protective antibodies. They could become immune against the virus in the short run [51], so these subjects may not personally rate interventions as critical. On the other hand, if a family member or a close friend is infected with the virus, the level of risk perception increases without this feeling of protection. It can explain why indirect experience triggers higher level of approval of the interventions connected to the higher risk perception, while direct experience does not.

## 5 Conclusion

In the last decades the use of nudges has become an essential part of the public policy toolbox in many countries of the world. Therefore, it was no surprise that they were also applied in the combat against the COVID-19 pandemic. Governments started to experiment with different forms of nudges to reach compliance with some non-pharmaceutical interventions such as mask wearing, hand hygiene, and social distancing. Various studies have reported about the high level of public support and the effectiveness of these nudges, but at the same time, some interventions proved to be less successful. This study looked at how risk perception and experience with COVID-19 influenced the approval of these policy interventions, and surfaces some practical implications for policy makers conducting risk communication campaigns.

The results of our study indicate that we should advise against the sole use of nudges in a pandemic that has brought unprecedented risks to most societies, since they suggest that the presence of high risk increases the public’s preference for stricter regulations. If governments underplay the seriousness of the pandemic, which would therefore make people perceive the level of risk lower, that would undermine the public acceptance of any policy measure. For this reason, politicians are in a delicate situation: they want to demonstrate their competencies and the effectiveness of the measures they implement, and they do not want to spark unnecessary panic. If the level of risk perception drops because of such a narrative, then citizens would not welcome the interventions needed. In contrast, a risk that is considered high, enhances the legitimacy of the introduced measures.

Since direct experience negatively influences policy support, policy makers should pay special attention to those who had already contracted COVID-19. They may diminish the public support of the preventive measures because of their selfish desire to get back more freedom, stemming from a feeling of immunity, or by spreading a message that the disease does not cause big trauma. As a pandemic develops, more and more people get infected, hence these interventions could be less and less supported because of this effect. Therefore, in their risk communication, authorities ought to emphasize the protection of family members, friends, and vulnerable members of society by complying with preventive measures, instead of underlining the inherent dangers of catching the virus.

Our study has some important limitations. The COVID-19 situation in all countries has been very dynamic, policies have often been introduced in short notice. These rapid changes influence significantly what respondents think about restrictions, mandates, information campaigns, and nudges. Due to the CAWI survey instrument that was used in this study, only people below the age of 64 were included into the sample, which excluded the most vulnerable portion of the population. The survey responses may also be slightly biased in absolute terms, since the items and question blocks were not randomised. An ordering effect may have increased support for policies because risk related questions were asked beforehand, and could have had an effect on the individual items as respondents can be expected to take the first item as a reference point. As the focus of our research questions was on relative differences between policy types and risk perception, not accounting for survey order effect should not weaken the findings. COVID-19 related topics have been heavily politicized in Hungary, therefore survey responses may, to some degree, depend on political preferences that were not investigated in the current study. People may embrace or reject nudge policies based on so-called partisan cues [52]; hence the role of preferences should be analyzed in future research.

## Supporting information

S1 Fig Development of COVID-19 in Hungary. The figure reports the course of the pandemic capturing two measures. The containment health index based on the data from Hale et al. [49] showing the strictness of policy measures and the number of daily new confirmed cases based on WHO data, smoothed with a seven day moving average.
(TIF)

S2 Fig Policy support of the preventive measures. The figure reports the percentage of respondents whose support was on the positive side of the scale (from ‘rather support’ to ‘certainly support’), the midpoint (neutral) not included. Error bars represent 95 percent confidence intervals.
(TIF)

S1 Data (DOCX)

## References

[1] Thaler RH, Sunstein CR. Nudge. New Haven & London: Yale University Press.; 2008.

[2] Szaszi B, Palinkas A, Palfi B, Szollosi A, Aczel B. A Systematic Scoping Review of the Choice Architecture Movement: Toward Understanding When and Why Nudges Work. Journal of Behavioral Decision Making. 2018;31(3):355–366. doi: 10.1002/bdm.2035

[3] Li M, Chapman GB. Nudge to Health: Harnessing Decision Research to Promote Health Behavior. Social and Personality Psychology Compass. 2013;7(3):187–198. doi: 10.1111/spc3.12019

[4] Schubert C. Green nudges: Do they work? Are they ethical? Ecological Economics. 2017;132:329–342. doi: 10.1016/j.ecolecon.2016.11.009

[5] Benartzi S, Thaler RH. Behavioral Economics and the Retirement Savings Crisis. Science. 2013;339(6124):1152–1153. doi: 10.1126/science.1231320 23471389

[6] Goldstein DG, Johnson EJ, Herrmann A, Heitmann M. Nudge Your Customers Toward Better Choices. Harvard Business Review. 2008;86(12):99–105.

[7] Fu Y, Ma W, Wu J. Fostering Voluntary Compliance in the COVID-19 Pandemic: An Analytical Framework of Information Disclosure. The American Review of Public Administration. 2020;50(6-7):685–691. doi: 10.1177/0275074020942102

[8] Franzen A, Wöhner F. Coronavirus risk perception and compliance with social distancing measures in a sample of young adults: Evidence from Switzerland. PLOS ONE. 2021;16(2):e0247447. doi: 10.1371/journal.pone.0247447 33606826 PMC7894933

[9] Sunstein CR. People Prefer System 2 Nudges (Kind of). Duke LJ. 2016;66:121–168.

[10] Sunstein CR, Reisch LA, Rauber J. A worldwide consensus on nudging? Not quite, but almost. Regulation & Governance. 2018;12(1):3–22. doi: 10.1111/rego.12161

[11] Diepeveen S, Ling T, Suhrcke M, Roland M, Marteau TM. Public acceptability of government intervention to change health-related behaviours: a systematic review and narrative synthesis. BMC Public Health. 2013;13(1):756. doi: 10.1186/1471-2458-13-756 23947336 PMC3765153

[12] Hagmann D, Siegrist M, Hartmann C. Taxes, labels, or nudges? Public acceptance of various interventions designed to reduce sugar intake. Food Policy. 2018;79:156–165. doi: 10.1016/j.foodpol.2018.06.008

[13] Quigley M. Nudging for health: on public policy and designing choice architecture. Medical Law Review. 2013;21(4):588–621. doi: 10.1093/medlaw/fwt022 24081425 PMC3818966

[14] Sunstein CR. The Ethics of Influence: Government in the Age of Behavioral Science. Cambridge University Press; 2016.

[15] Hagmann D, Ho EH, Loewenstein G. Nudging out support for a carbon tax. Nature Climate Change. 2019;9(6):484–489. doi: 10.1038/s41558-019-0474-0

[16] Benartzi S, Beshears J, Milkman KL, Sunstein CR, Thaler RH, Shankar M, et al. Should Governments Invest More in Nudging? Psychological Science. 2017;28(8):1041–1055. doi: 10.1177/0956797617702501 28581899 PMC5549818

[17] Sunstein CR, Reisch LA, Kaiser M. Trusting nudges? Lessons from an international survey. Journal of European Public Policy. 2019;26(10):1417–1443. doi: 10.1080/13501763.2018.1531912

[18] Jung JY, Mellers BA. American attitudes toward nudges. Judgment and Decision Making. 2016;11(1):62–74.

[19] Reynolds JP, Pilling M, Marteau TM. Communicating quantitative evidence of policy effectiveness and support for the policy: Three experimental studies. Social Science & Medicine. 2018;218:1–12. doi: 10.1016/j.socscimed.2018.09.037 30312911

[20] Sunstein CR. Nudges vs. Shoves. Harvard Law Review Forum. 2013;127:210.

[21] Hansen PG, Jespersen AM. Nudge and the Manipulation of Choice: A Framework for the Responsible Use of the Nudge Approach to Behaviour Change in Public Policy. European Journal of Risk Regulation. 2013;4(1):3–28. doi: 10.1017/S1867299X00002762

[22] Felsen G, Castelo N, Reiner PB. Decisional enhancement and autonomy: public attitudes towards overt and covert nudges. Judgment and Decision Making. 2013;8(3):202.

[23] Hagman W, Andersson D, Västfjäll D, Tinghög G. Public Views on Policies Involving Nudges. Review of Philosophy and Psychology. 2015;6(3):439–453. doi: 10.1007/s13164-015-0263-2

[24] Reisch LA, Sunstein CR, Gwozdz W. Viewpoint: Beyond carrots and sticks: Europeans support health nudges. Food Policy. 2017;69:1–10. doi: 10.1016/j.foodpol.2017.01.007

[25] Junghans AF, Cheung TT, De Ridder DD. Under consumers’ scrutiny—an investigation into consumers’ attitudes and concerns about nudging in the realm of health behavior. BMC Public Health. 2015;15(1):336. doi: 10.1186/s12889-015-1691-8 25881161 PMC4393866

[26] Pe’er E, Feldman Y, Gamliel E, Sahar L, Tikotsky A, Hod N, et al. Do minorities like nudges? The role of group norms in attitudes towards behavioral policy. Judgment and Decision Making. 2019;14(1):40–50.

[27] Reisch LA, Sunstein CR. Do Europeans Like Nudges? Judgment and Decision making. 2016;11(4):310–325.

[28] Bavel JJV, Baicker K, Boggio PS, Capraro V, Cichocka A, Cikara M, et al. Using social and behavioural science to support COVID-19 pandemic response. Nature Human Behaviour. 2020;4(5):460–471. doi: 10.1038/s41562-020-0884-z 32355299

[29] Capraro V, Boggio P, Böhm R, Perc M, Sjåstad H. Cooperation and acting for the greater good during the COVID-19 pandemic. PsyArXiv; 2021. Available from: https://psyarxiv.com/65xmg/.

[30] Weijers RJ, de Koning BB. Nudging to increase hand hygiene during the COVID-19 pandemic: A field experiment. Canadian Journal of Behavioural Science / Revue canadienne des sciences du comportement. 2020.

[31] Guan B, Bao G, Liu Q, Raymond RG. Two-Way Risk Communication, Public Value Consensus, and Citizens’ Policy Compliance Willingness About COVID-19: Multilevel Analysis Based on Nudge View. Administration & Society. 2021. doi: 10.1177/0095399721990332

[32] Moriwaki D, Harada S, Schneider J, Hoshino T. Nudging Preventive Behaviors in COVID-19 Crisis: A Large Scale RCT using Smartphone Advertising. Institute for Economics Studies, Keio University; 2020. 2020-021. Available from: https://ideas.repec.org/p/keo/dpaper/2020-021.html.

[33] Prasetyo DB, Sofyan L. Altering Intention to Mudik during COVID-19 Pandemic: A Salient Cue and Simple Reminder Treatment. Available at SSRN 3595007. 2020.

[34] Debnath R, Bardhan R. India nudges to contain COVID-19 pandemic: A reactive public policy analysis using machine-learning based topic modelling. PLOS ONE. 2020;15(9):e0238972. doi: 10.1371/journal.pone.0238972 32915899 PMC7485898

[35] Hume S, John P, Sanders M, Stockdale E. Nudge in the Time of Coronavirus: The Compliance to Behavioural Messages during Crisis. Available at SSRN 3644165. 2020.

[36] Blackman A, Hoffmann B. Diminishing Returns: Nudging Covid-19 Prevention Among Colombian Young Adults. IDB Working Paper Series; 2021. 1217. 10.1371/journal.pone.0279179 PMC9778522 36548257

[37] Banker S, Park J. Evaluating Prosocial COVID-19 Messaging Frames: Evidence from a Field Study on Facebook. Judgment and Decision Making. 2020;15(6):1037–1043.

[38] Capraro V, Barcelo H. The effect of messaging and gender on intentions to wear a face covering to slow down COVID-19 transmission. Journal of Behavioral Economics for Policy. 2020;4(S2):45–55.

[39] Sibony AL. The UK COVID-19 Response: A Behavioural Irony? European Journal of Risk Regulation. 2020;11(2):350–357. doi: 10.1017/err.2020.22

[40] Tummers L. Public Policy and Behavior Change. Public Administration Review. 2019;79(6):925–930. doi: 10.1111/puar.13109

[41] Drews S, Bergh JCJMvd. What explains public support for climate policies? A review of empirical and experimental studies. Climate Policy. 2016;16(7):855–876. doi: 10.1080/14693062.2015.1058240

[42] Zahran S, Brody SD, Grover H, Vedlitz A. Climate Change Vulnerability and Policy Support. Society & Natural Resources. 2006;19(9):771–789. doi: 10.1080/08941920600835528

[43] Liu X, Mumpower JL, Portney KE, Vedlitz A. Perceived Risk of Terrorism and Policy Preferences for Government Counterterrorism Spending: Evidence From a U.S. National Panel Survey. Risk, Hazards & Crisis in Public Policy. 2019;10(1):102–135. doi: 10.1002/rhc3.12154

[44] Gerber BJ, Neeley GW. Perceived Risk and Citizen Preferences for Governmental Management of Routine Hazards. Policy Studies Journal. 2005;33(3):395–418. doi: 10.1111/j.1541-0072.2005.00122.x

[45] Bates S, Holmes J, Gavens L, de Matos EG, Li J, Ward B, et al. Awareness of alcohol as a risk factor for cancer is associated with public support for alcohol policies. BMC Public Health. 2018;18(1):688. doi: 10.1186/s12889-018-5581-8 29866082 PMC5987582

[46] Dryhurst S, Schneider CR, Kerr J, Freeman ALJ, Recchia G, van der Bles AM, et al. Risk perceptions of COVID-19 around the world. Journal of Risk Research. 2020;23(7-8):994–1006. doi: 10.1080/13669877.2020.1758193

[47] van der Linden S. On the relationship between personal experience, affect and risk perception: The case of climate change. European Journal of Social Psychology. 2014;44(5):430–440. doi: 10.1002/ejsp.2008 25678723 PMC4312984

[48] Purnell JQ, Thompson T, Kreuter MW, McBride TD. Behavioral economics: “nudging” underserved populations to be screened for cancer. Preventing Chronic Disease. 2015;12:E06. doi: 10.5888/pcd12.140346 25590600 PMC4307834

[49] Hale T, Angrist N, Goldszmidt R, Kira B, Petherick A, Phillips T, et al. A global panel database of pandemic policies (Oxford COVID-19 Government Response Tracker). Nature Human Behaviour. 2021;5(4):529–538. doi: 10.1038/s41562-021-01079-8 33686204

[50] Kasperson RE, Renn O, Slovic P, Brown HS, Emel J, Goble R, et al. The Social Amplification of Risk: A Conceptual Framework. Risk Analysis. 1988;8(2):177–187. doi: 10.1111/j.1539-6924.1988.tb01168.x

[51] Altmann DM, Douek DC, Boyton RJ. What policy makers need to know about COVID-19 protective immunity. The Lancet. 2020;395(10236):1527–1529. doi: 10.1016/S0140-6736(20)30985-5 32353328 PMC7185915

[52] Tannenbaum D, Fox CR, Rogers T. On the misplaced politics of behavioural policy interventions. Nature Human Behaviour. 2017;1(7):1–7. doi: 10.1038/s41562-017-0130

