# Supplementary figures and images for: Nudging in the time of coronavirus? Comparing public support for soft and hard preventive measures, highlighting the role of risk perception and experience
Source: PLoS One. 2021 Aug 13;16(8):e0256241. doi: 10.1371/journal.pone.0256241 (PMC8362989; doi:10.1371/journal.pone.0256241)

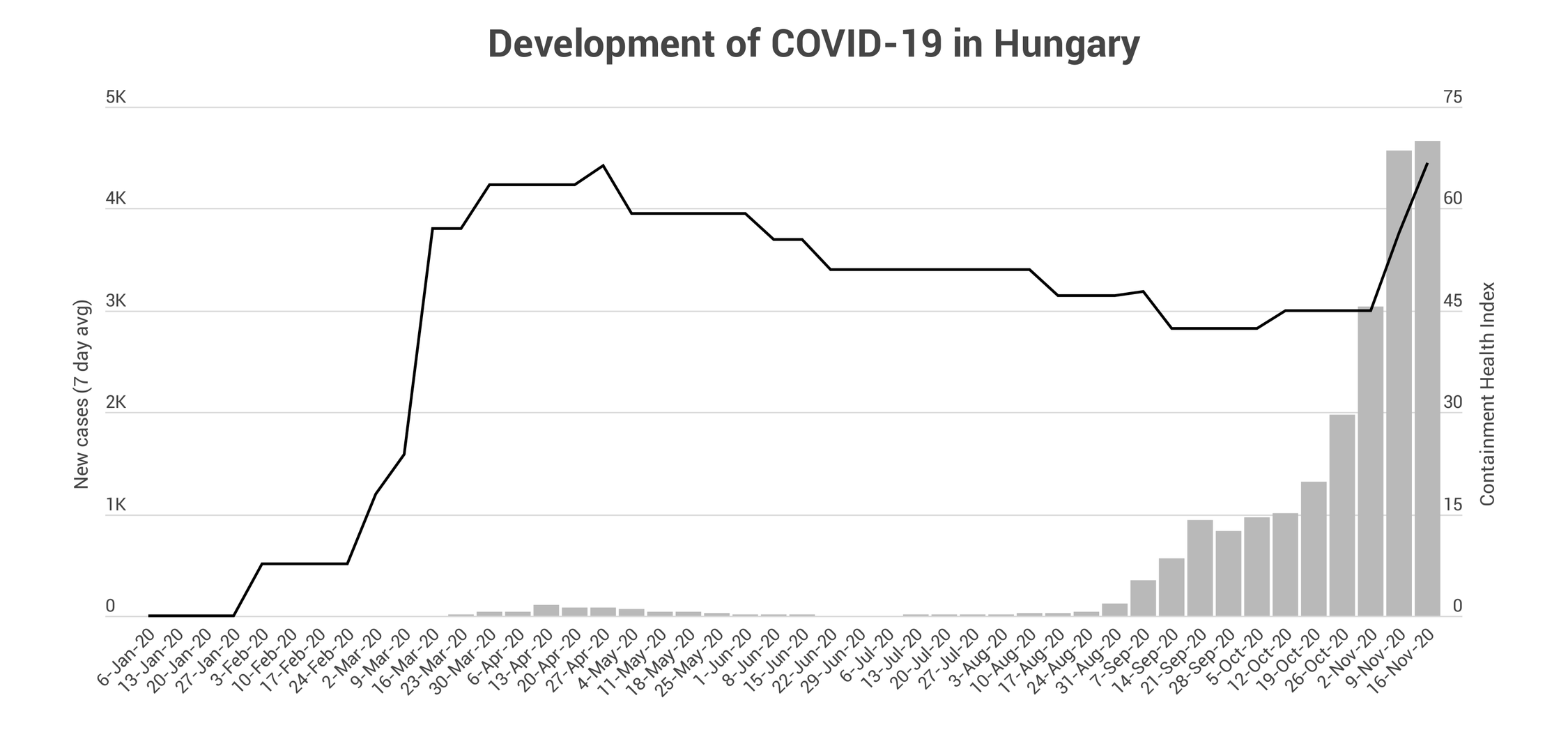

Supplement: S1 Fig — The figure reports the course of the pandemic capturing two measures. The containment health index based on the data from Hale et al. [49] showing the strictness of policy measures and the number of daily new confirmed cases based on WHO data, smoothed with a seven day moving average. (TIF) [file pone.0256241.s001.tif]

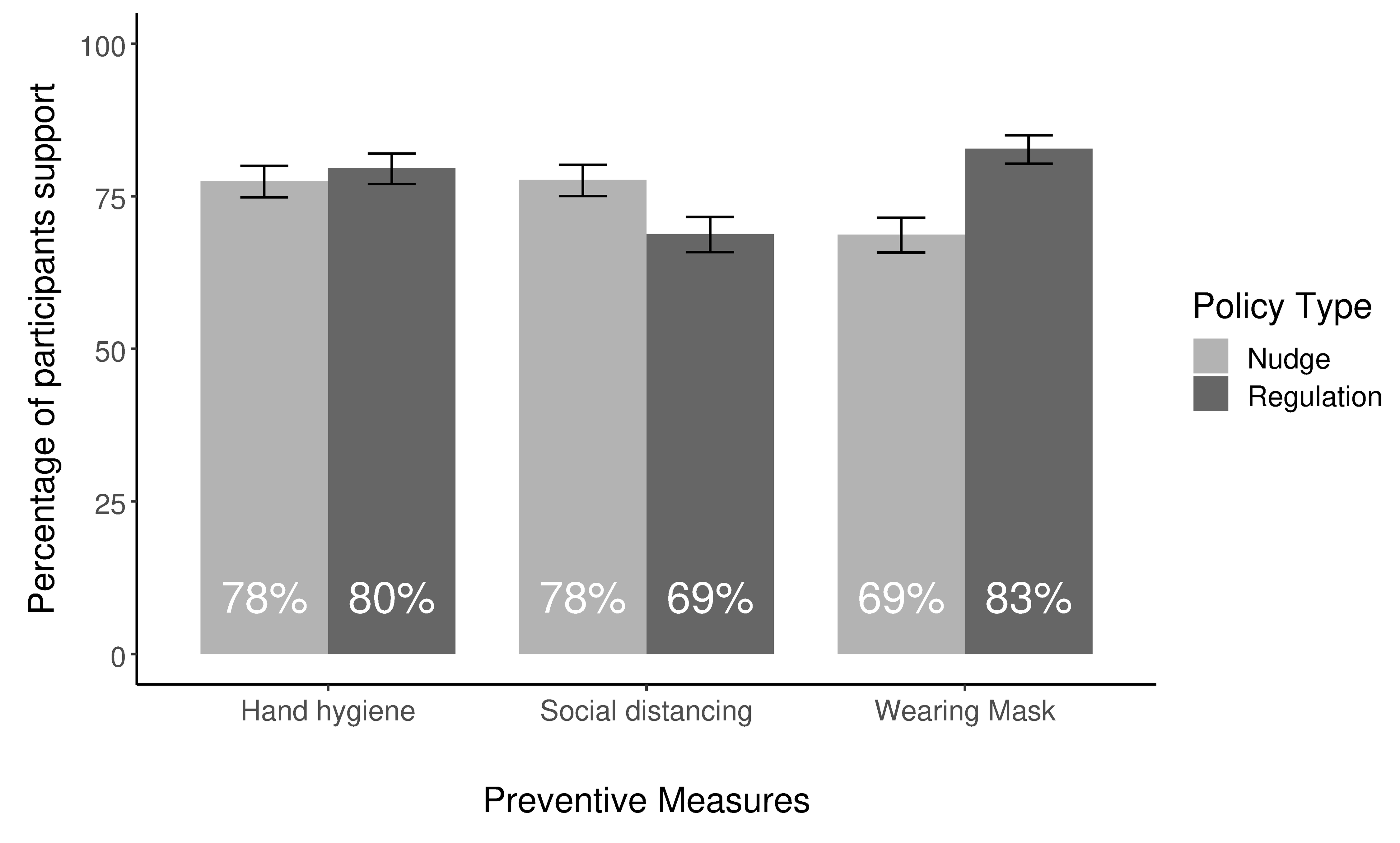

Supplement: S2 Fig — The figure reports the percentage of respondents whose support was on the positive side of the scale (from ‘rather support’ to ‘certainly support’), the midpoint (neutral) not included. Error bars represent 95 percent confidence intervals. (TIF) [file pone.0256241.s002.tif]
