# Supplementary material for: Nudging in the time of coronavirus? Comparing public support for soft and hard preventive measures, highlighting the role of risk perception and experience
Source: PLoS One. 2021 Aug 13;16(8):e0256241. doi: 10.1371/journal.pone.0256241 (PMC8362989; doi:10.1371/journal.pone.0256241)
Supplement: S1 Data — (DOCX) [file pone.0256241.s003.docx]

**S1 Data.**

Further supporting data are also publicly available on the Open Science Framework (OSF) website: https://osf.io/4637w/. The repository contains: full survey questionnaire, raw survey data, list of variables (codebook) and scripts of the analysis (R code). DOI: 10.17605/OSF.IO/4637W
