# Supplementary material for: Nudging in the time of coronavirus? Comparing public support for soft and hard preventive measures, highlighting the role of risk perception and experience
Source: PLoS One. 2021 Aug 13;16(8):e0256241. doi: 10.1371/journal.pone.0256241 (PMC8362989; doi:10.1371/journal.pone.0256241)
Supplement: Attachment — Submitted filename: Response to Reviewers.pdf [file pone.0256241.s004.pdf]

Dear Editor and Reviewers,

Thank you for your kind and valuable feedback. The authors of this paper made an effort to adhere to all the comments and criticism. Below we provide an answer for all raised issues. Please find the author's responses as the blue colored text.

EDITORS feedback

1. Please ensure that your manuscript meets PLOS ONE's style requirements, including those for file naming.

Authors reply:

Thanks for the notice. Updated supplementary figure naming in the text and at the end and also made sure to provide proper file names during revision. We are using PLOS ONE latex template, and happy to make any further adjustments if necessary.

2. Thank you for including your ethics statement:

"All participants provided informed consent. The research

has received all relevant university ethics approval".

a) Please amend your current ethics statement to include the full name of the ethics committee/institutional review board(s) that approved your specific study.

Authors reply:

Included name of ethics committe in the manuscript.

b) Please provide additional details regarding participant consent. In the ethics statement in the Methods and online submission information, please ensure that you have specified what type you obtained (for instance, written or verbal, and if verbal, how it was documented and witnessed). If your study included minors, state whether you obtained consent from parents or guardians. If the need for consent was waived by the ethics committee, please include this information.

Authors reply:

Augmented the refernce on consent ('written'). The omnibus company we employed for data collection is operating with the highest of standards (European guidelines, GDPR), when it comes to data collection and handling. We can provide more information if needed (but documentation is mainly in Hungarian)

Once you have amended this/these statement(s) in the Methods section of the manuscript, please add the same text to the "Ethics Statement" field of the submission form (via "Edit Submission").

Authors reply:

Updated in submission as well

3. We note you have included a table to which you do not refer in the text of your manuscript. Please ensure that you refer to Table 4 in your text; if accepted, production will need this reference to link the reader to the Table.

Authors reply:

Included a sentence in the Results & Discussion section about the table, with appropriate reference.

4. Please include captions for your Supporting Information files at the end of your manuscript, and update any in-text citations to match accordingly.

Authors reply:

Revised the reference to the figure namings (included as required in the supplementary materials), and augmented with label

REVIEWER #1

1.1 Context: COVID prevention measures being implemented by the government to reduce infections. Therefore, absolute and relative support is not independent of the context. From the paper, we don't learn much about conditional effects (e.g., by ideology, support of the government, etc.) that have already been explored in the literature. All we learn is that in a context in which the government is telling the population that strict measures are

needed to curve COVID, the average individual in the sample is more in favor if she perceives the risk to be higher.

Authors reply:

It is a fair point that contextual factors influence the relative support of nudges and regulations. There are numerous prior studies dealing with these effects, and some of them are mentioned in the present study. Nonetheless, the focus of this study was to investigate the role of risk perception and the direct and indirect experience on public support. One must also consider that questions about party preferences and ideologies have been very sensitive in the Hungarian context. COVID-19 issues have been highly politicized, and they are potential hot topics of the incoming elections. Therefore, we decided not to analyze the role of these contextual factors. However, in future research these are absolutely valid directions.

1.2. Method: the authors ran a survey of 1,000 individuals in Hungary (more about the sample below). They asked all of them the whole set of questions. It does not seem that they randomized the order the questions were presented. Therefore, one question may be priming responses for the rest, and responses may be correlated (once you ask for the first measure, the respondents adjust their subsequent responses to the first to show consistency), particularly given the context in which the survey took place. Given the context, the authors should have randomized the ordering of the questions. They could have randomized individuals into groups and assign them to vignettes or information to better identify the effect of external factors on their responses.

Authors reply:

We included a few sentences about this caveat in the limitations paragraph (end of Conclusion section) because it is a valid concern that not randomising does not correct for any order effect that may have had an effect on the respondents. We argue that this may have affected the results in absolute terms, but it is unlikely to affect the relative differences significantly. As the focus of our research questions was on relative differences between support for nudges and regulation; and support from people with lower or higher levels of perceived risk, not accounting for survey order effect does not weaken our findings.

1.3. The authors motivate their paper and spend some time discussing the literature on support for nudges. They set up their analysis to compare the relative support for nudges vis-a-vis regulations. First, it is difficult to isolate underlying support given the context. Second, while for the average individual is intuitive to understand the effect of regulations, it is not necessarily the case regarding the effect of a nudge. Not so long ago, academics and policymakers could not have predicted that defaults mattered, that anchoring is a thing, or that individuals use “system 1” or “system 2” when making decisions. Therefore, assuming that respondents are equally capable and knowledgeable about the different policy alternatives is not credible.

Authors reply:

Nudges in this study were carefully selected in order to assure that people understand the aim and the relevance of these soft policies. Nudges in questions were introduced to the Hungarian society earlier that year, and they became part of people’s everyday life. Hence, we can assume that the rationale behind these nudges were understood by the majority of the Hungrian citizens. Of course, we must admit that there still might have been some people who did not understand the effect of nudges, but other studies revealed that by disclosing the purpose or the underlying mechanisms of nudges, does not change the level of acceptance or the effectiveness of these behavioral policies (Loewenstein et al., 2015; Kroese et al., 2016; Bruns et al., 2018).

2. Edit the document. The document becomes repetitive, and sometimes is difficult to assess the main messages.

Authors reply:

To create a more streamline flow for the readers, the document was thoroughly restructured. We split the Introduction and introduced a Backgrounds section, written a Statistical analysis subsection of the Methods to outline the performed analysis logic, and make the results section more easily interpretable. Combined the Results and Discussion sections to eliminate repetitiveness and close the paper with a partly new Conclusions section focusing on the main policy messages.

3. The authors argue that the sample is representative of the Hungarian population in terms of age, gender, and education. This claim is not proven. To start with, by construction, the sample is younger than the overall population. Second, the data was collected online, which by definition implies self-selection into the sample. Third, the sample has a higher share of females than what the demographics of the country indicate. Therefore, the authors should provide demographics information and describe any deviations that the sample has for external validity.

Authors reply:

Very fair point that more information about the sample composition would be prudent to disclose. For this reason we included a new table detailing information about the sample, bucketed by the three criteria we list for representative sampling. Also included in the main text the caveat of the sample being truly representative of the Hungarian population: namely the lack of participants above 64 (hence the younger average age the reviewer noted), and the CAWI nature of the survey likely introducing a bias.

As for the reviewers comment on the distribution between the sexes, our sample is representative of the Hungarian population between 18-64 according to the data of Central Statistics Office (KSH) of Hungary.

REVIEWER #1

Thank you for your submission to PLOS One. The paper is very interesting and important from a behavioural public policy lens in pandemic management. In general, I think the paper is academically sound, however, I think the structuring needs a major revision. Please find my section specific comments:

1. Abstract is good, can be better if you reflect the analytical results in the last three sentences.

Authors reply:

Thanks you. We included a short notes to reference analytical results in the abstract as well.

2. Introduction section is well-written and stands good as it is. instead of breaking the introduction in four subsections. I would suggest your add a section 2 called background, and place the subsection 1.1 - 1.4.

Authors reply:

Thanks for the good suggestion, we introduced Backgrounds section

In the background section, please add some more instances of Hungarian context and its policy response. I think it is very important for the readers to understand the context well, as the outcome of nudging behaviour is dependent on its local contexts. A figure showing how government responded to COVID-19 via nudges (soft and hard) will be very informative.

Authors reply:

A new subsection of Backgrounds (2.5) and a new figure S1 provides more context on the Hungarian development of COVID-19

3. In the same section, try to reflect the diversity of nudging approaches already used in pandemic management. I saw you mentioned the case of the UK, but more examples will build a compelling story. Some of the papers that I found interesting are:

https://bjsm.bmj.com/content/54/20/1183.abstract

https://publications.iadb.org/publications/english/document/Diminishing-Returns-Nudging-Covid-19-Prevention-Among-Colombian-Young-Adults.pdf (Working paper)

https://www.ncbi.nlm.nih.gov/pmc/articles/PMC7182304/

https://ideas.repec.org/p/keo/dpaper/2020-021.html (Working paper)

https://journals.plos.org/plosone/article?id=10.1371/journal.pone.0238972

Authors reply:

Thank you, for the very interesting articles! We slightly revised the section (2.2) detailing nudges used during the pandemic, introducing the distinction of which studies support their effectiveness and which studies do not. As a result removed one reference that had no empirical results (Park 2020)

From the five advised articles, we also included three but, Huyn (2020), was not referenced in this section because it also does not report about empirical findings. We did not refer to Ding et al. (2020) because their use of word 'nudge' is not consistent with the "thalerian" definition we rely on - the lockdown is a much more intrusive phenomenon, taking away, and completely changing possibilities.

4. Section 2: Material and method. I think this section needs a major restructuring as I saw you mentioned all the statistical tests in the results section. Please arrange it and create a streamline flow of the methodology you mentioned separately in the results section. I think best will be to add a section '2.3 Statistical analysis/quantitative analysis'

Authors reply:

Thank you for the good advice on the structure, we have written statistical analysis subsection (3.3)

5. Following from my previous comments, please combine results and discussion section. As I found your current discussion section to be full with references. While this is acceptable, but I think you need to moderate the reference to specifically support your quantitative evidence.

Authors reply:

Again, good advice adhered to. Removed repetitiveness by combining the two sections.

6. Please add a conclusion section, and try to focus on a key policy message. In the introduction you were critical about nudging as a policy response and that should be reflected again in a conclusion section so that the readers understand the value of behavioural public policy measures.

Authors reply:

We included a Conclusion section focusing on the key policy messages
