# Supplementary material for: Nudging in the time of coronavirus? Comparing public support for soft and hard preventive measures, highlighting the role of risk perception and experience
Source: PLoS One. 2021 Aug 13;16(8):e0256241. doi: 10.1371/journal.pone.0256241 (PMC8362989; doi:10.1371/journal.pone.0256241)
Supplement: Attachment — Submitted filename: Response to Reviewers - plos one v2.pdf [file pone.0256241.s005.pdf]

Response to Reviewers
PONE-D-21-12439R1
2nd revision - invite for minor revision

Response to Reviewer 2.

a) writing style of section 2.1 to 2.3 should maintain neutrality to respect the papers that you have cited.

Author response: thank you for noticing, we agree the tonality should maintain neutrality and for this reason we rephrased some of our remarks on the cited articles

b) figures should be included in the main text, not relegated to appendix

Author response: we omitted the figures from the main text as per *PLOS* ONE submission guidelines. We agree they should be included in the main text in the published form of the paper, and noted their place in the manuscript for reference to reviewers and the publication production office.

https://journals.plos.org/plosone/s/submission-guidelines requires:
*"Do not include figures in the main manuscript file. Each figure must be prepared and submitted as an individual file."*

c) improve the writing of the conclusion section

Author response: the sentences in the conclusion section were adjusted to improve the writing.

Response to Editor

a) the literature review is quite incomplete. I think you should make an effort to review the literature in the best possible ways.

Author response: the literature review were extended and the three mentioned articles were included in section 2.2
