# Supplementary material for: Nudging in the time of coronavirus? Comparing public support for soft and hard preventive measures, highlighting the role of risk perception and experience
Source: PLoS One. 2021 Aug 13;16(8):e0256241. doi: 10.1371/journal.pone.0256241 (PMC8362989; doi:10.1371/journal.pone.0256241)
Supplement: Peer review history

# Peer review: Decision Letter 0

**DOI:** 10.1371/journal.pone.0256241.r001

**Authors:** Valerio Capraro

18 Jun 2021

PONE-D-21-12439

Nudging in the time of coronavirus? Comparing public support for soft and hard preventive measures, highlighting the role of risk perception and experience

PLOS ONE

Dear Dr. Dudás,

Thank you for submitting your manuscript to PLOS ONE. After careful consideration, we feel that it has merit but does not fully meet PLOS ONE’s publication criteria as it currently stands. Therefore, we invite you to submit a revised version of the manuscript that addresses the points raised during the review process.

Please find below the reviewers' comments as well as those of mine.

Please submit your revised manuscript by Aug 02 2021 11:59PM. If you will need more time than this to complete your revisions, please reply to this message or contact the journal office at plosone@plos.org. When you're ready to submit your revision, log on to https://www.editorialmanager.com/pone/ and select the 'Submissions Needing Revision' folder to locate your manuscript file.

Please include the following items when submitting your revised manuscript:

- A rebuttal letter that responds to each point raised by the academic editor and reviewer(s). You should upload this letter as a separate file labeled 'Response to Reviewers'.
- A marked-up copy of your manuscript that highlights changes made to the original version. You should upload this as a separate file labeled 'Revised Manuscript with Track Changes'.
- An unmarked version of your revised paper without tracked changes. You should upload this as a separate file labeled 'Manuscript'.

If you would like to make changes to your financial disclosure, please include your updated statement in your cover letter. Guidelines for resubmitting your figure files are available below the reviewer comments at the end of this letter.

If applicable, we recommend that you deposit your laboratory protocols in protocols.io to enhance the reproducibility of your results. Protocols.io assigns your protocol its own identifier (DOI) so that it can be cited independently in the future. For instructions see: http://journals.plos.org/plosone/s/submission-guidelines#loc-laboratory-protocols. Additionally, PLOS ONE offers an option for publishing peer-reviewed Lab Protocol articles, which describe protocols hosted on protocols.io. Read more information on sharing protocols at https://plos.org/protocols?utm_medium=editorial-email&utm_source=authorletters&utm_campaign=protocols.

We look forward to receiving your revised manuscript.

Kind regards,

Valerio Capraro

Academic Editor

PLOS ONE

Journal Requirements:

When submitting your revision, we need you to address these additional requirements.

1. Please ensure that your manuscript meets PLOS ONE's style requirements, including those for file naming. The PLOS ONE style templates can be found at

https://journals.plos.org/plosone/s/file?id=wjVg/PLOSOne_formatting_sample_main_body.pdf and

https://journals.plos.org/plosone/s/file?id=ba62/PLOSOne_formatting_sample_title_authors_affiliations.pdf

2.  Thank you for including your ethics statement: 

"All participants provided informed consent. The research

has received all relevant university ethics approval".   

a) Please amend your current ethics statement to include the full name of the ethics committee/institutional review board(s) that approved your specific study.

b) Please provide additional details regarding participant consent. In the ethics statement in the Methods and online submission information, please ensure that you have specified what type you obtained (for instance, written or verbal, and if verbal, how it was documented and witnessed). If your study included minors, state whether you obtained consent from parents or guardians. If the need for consent was waived by the ethics committee, please include this information.

Once you have amended this/these statement(s) in the Methods section of the manuscript, please add the same text to the “Ethics Statement” field of the submission form (via “Edit Submission”).

For additional information about PLOS ONE ethical requirements for human subjects research, please refer to http://journals.plos.org/plosone/s/submission-guidelines#loc-human-subjects-research.

3. We note you have included a table to which you do not refer in the text of your manuscript. Please ensure that you refer to Table 4 in your text; if accepted, production will need this reference to link the reader to the Table.

4. Please include captions for your Supporting Information files at the end of your manuscript, and update any in-text citations to match accordingly. Please see our Supporting Information guidelines for more information: http://journals.plos.org/plosone/s/supporting-information.

[Note: HTML markup is below. Please do not edit.]

Reviewers' comments:

Reviewer's Responses to Questions

**Comments to the Author**

1. Is the manuscript technically sound, and do the data support the conclusions?

The manuscript must describe a technically sound piece of scientific research with data that supports the conclusions. Experiments must have been conducted rigorously, with appropriate controls, replication, and sample sizes. The conclusions must be drawn appropriately based on the data presented. 

Reviewer #1: Partly

Reviewer #2: Yes

**********

2. Has the statistical analysis been performed appropriately and rigorously? 

Reviewer #1: Yes

Reviewer #2: Yes

**********

3. Have the authors made all data underlying the findings in their manuscript fully available?

The PLOS Data policy requires authors to make all data underlying the findings described in their manuscript fully available without restriction, with rare exception (please refer to the Data Availability Statement in the manuscript PDF file). The data should be provided as part of the manuscript or its supporting information, or deposited to a public repository. For example, in addition to summary statistics, the data points behind means, medians and variance measures should be available. If there are restrictions on publicly sharing data—e.g. participant privacy or use of data from a third party—those must be specified.

Reviewer #1: Yes

Reviewer #2: Yes

**********

4. Is the manuscript presented in an intelligible fashion and written in standard English?

PLOS ONE does not copyedit accepted manuscripts, so the language in submitted articles must be clear, correct, and unambiguous. Any typographical or grammatical errors should be corrected at revision, so please note any specific errors here.

Reviewer #1: Yes

Reviewer #2: No

**********

5. Review Comments to the Author

Please use the space provided to explain your answers to the questions above. You may also include additional comments for the author, including concerns about dual publication, research ethics, or publication ethics. (Please upload your review as an attachment if it exceeds 20,000 characters)

Reviewer #1: General comments

The document attempts to assess public support for preventive measures during COVID-19, social distancing, hand hygiene, and wearing masks, for two sets of policy tools commonly used by policymakers: nudges and regulations. The authors evaluate whether support for either is affected by respondents’ risk perception and experience with the disease.

The authors exploit a survey of 1000 individuals in Hungary. “People with a higher level of risk perception supported both types of policies more but slightly favored the regulations. Those who had contact with the disease (either themselves or a close friend or family member contracting COVID-19) reported a higher level of risk perception. When the person themselves was afflicted, this higher levels of risk perception did not translate to a higher level of support, moreover, it even decreased support for the regulations. In case of a loved one contracting the disease, there was an increased support for both types of measures, consequent of the higher risk perception.”

While the authors made a good effort collecting the data, the survey does not isolate contextual factors, and it is not designed in a way that would help to provide novel insights (for example, if the authors had randomized assignment to different vignettes, we might have been able to learn how individuals react to an exogenous factor when answering).

Therefore, the paper results indicate that during a second wave of COVID-19, once the government had implemented strict regulatory measures to curve infections, those individuals who perceived that risk was higher showed higher support for those restrictive measures.

Specific comments

1. The authors attempt to evaluate the support for preventive measures. In particular, they are interested to assess whether or not the population had differential support for traditional regulatory measures compared to nudges.

There are several problems with that:

1.1 Context: COVID prevention measures being implemented by the government to reduce infections. Therefore, absolute and relative support is not independent of the context. From the paper, we don’t learn much about conditional effects (e.g., by ideology, support of the government, etc.) that have already been explored in the literature. All we learn is that in a context in which the government is telling the population that strict measures are needed to curve COVID, the average individual in the sample is more in favor if she perceives the risk to be higher.

1.2. Method: the authors ran a survey of 1,000 individuals in Hungary (more about the sample below). They asked all of them the whole set of questions. It does not seem that they randomized the order the questions were presented. Therefore, one question may be priming responses for the rest, and responses may be correlated (once you ask for the first measure, the respondents adjust their subsequent responses to the first to show consistency), particularly given the context in which the survey took place. Given the context, the authors should have randomized the ordering of the questions. They could have randomized individuals into groups and assign them to vignettes or information to better identify the effect of external factors on their responses.

1.3. The authors motivate their paper and spend some time discussing the literature on support for nudges. They set up their analysis to compare the relative support for nudges vis-a-vis regulations. First, it is difficult to isolate underlying support given the context. Second, while for the average individual is intuitive to understand the effect of regulations, it is not necessarily the case regarding the effect of a nudge. Not so long ago, academics and policymakers could not have predicted that defaults mattered, that anchoring is a thing, or that individuals use “system 1” or “system 2” when making decisions. Therefore, assuming that respondents are equally capable and knowledgeable about the different policy alternatives is not credible.

2. Edit the document. The document becomes repetitive, and sometimes is difficult to assess the main messages.

3. The authors argue that the sample is representative of the Hungarian population in terms of age, gender, and education. This claim is not proven. To start with, by construction, the sample is younger than the overall population. Second, the data was collected online, which by definition implies self-selection into the sample. Third, the sample has a higher share of females than what the demographics of the country indicate. Therefore, the authors should provide demographics information and describe any deviations that the sample has for external validity.

Reviewer #2: Thank you for your submission to PLOS One. The paper is very interesting and important from a behavioural public policy lens in pandemic management. In general, I think the paper is academically sound, however, I think the structuring needs a major revision. Please find my section specific comments:

1. Abstract is good, can be better if you reflect the analytical results in the last three sentences.

2. Introduction section is well-written and stands good as it is. instead of breaking the introduction in four subsections. I would suggest your add a section 2 called background, and place the subsection 1.1 - 1.4. In the background section, please add some more instances of Hungarian context and its policy response. I think it is very important for the readers to understand the context well, as the outcome of nudging behaviour is dependent on its local contexts. A figure showing how government responded to COVID-19 via nudges (soft and hard) will be very informative.

3. In the same section, try to reflect the diversity of nudging approaches already used in pandemic management. I saw you mentioned the case of the UK, but more examples will build a compelling story. Some of the papers that I found interesting are:

https://bjsm.bmj.com/content/54/20/1183.abstract

https://publications.iadb.org/publications/english/document/Diminishing-Returns-Nudging-Covid-19-Prevention-Among-Colombian-Young-Adults.pdf (Working paper)

https://www.ncbi.nlm.nih.gov/pmc/articles/PMC7182304/

https://ideas.repec.org/p/keo/dpaper/2020-021.html (Working paper)

https://journals.plos.org/plosone/article?id=10.1371/journal.pone.0238972

4. Section 2: Material and method. I think this section needs a major restructuring as I saw you mentioned all the statistical tests in the results section. Please arrange it and create a streamline flow of the methodology you mentioned separately in the results section. I think best will be to add a section '2.3 Statistical analysis/quantitative analysis'

5. Following from my previous comments, please combine results and discussion section. As I found your current discussion section to be full with references. While this is acceptable, but I think you need to moderate the reference to specifically support your quantitative evidence.

6. Please add a conclusion section, and try to focus on a key policy message. In the introduction you were critical about nudging as a policy response and that should be reflected again in a conclusion section so that the readers understand the value of behavioural public policy measures.

**********

6. PLOS authors have the option to publish the peer review history of their article (what does this mean?). If published, this will include your full peer review and any attached files.

If you choose “no”, your identity will remain anonymous but your review may still be made public.

**Do you want your identity to be public for this peer review?** For information about this choice, including consent withdrawal, please see our Privacy Policy.

Reviewer #1: No

Reviewer #2: No

[NOTE: If reviewer comments were submitted as an attachment file, they will be attached to this email and accessible via the submission site. Please log into your account, locate the manuscript record, and check for the action link "View Attachments". If this link does not appear, there are no attachment files.]

While revising your submission, please upload your figure files to the Preflight Analysis and Conversion Engine (PACE) digital diagnostic tool, https://pacev2.apexcovantage.com/. PACE helps ensure that figures meet PLOS requirements. To use PACE, you must first register as a user. Registration is free. Then, login and navigate to the UPLOAD tab, where you will find detailed instructions on how to use the tool. If you encounter any issues or have any questions when using PACE, please email PLOS at figures@plos.org. Please note that Supporting Information files do not need this step.

# Author comment: Author response to Decision Letter 0

**DOI:** 10.1371/journal.pone.0256241.r002

9 Jul 2021

Dear Editor and Reviewers,

Thank you for your kind and valuable feedback. The authors of this paper made an effort to adhere to all the comments and criticism. Below we provide an answer for all raised issues. 

For the EDITOR

1. 

Thanks for the notice. Updated supplementary figure naming in the text and at the end and also made sure to provide proper file names during revision. We are using PLOS ONE latex template, and happy to make any further adjustments if necessary.

2. a) 

Included name of ethics committe in the manuscript (section 3.1) and the Ethics Statement as well.

2. b) 

Augmented the refernce on consent (‘written’). The omnibus company we employed for data collection is operating with the highest of standards (European guidelines, GDPR), when it comes to data collection and handling. We can provide more information if needed (but documentation is mainly in Hungarian). Updated in submission as well

3. 

Included a sentence in the Results & Discussion section about the table, with appropriate reference.

4. 

Revised the reference to the figure namings (included as required in the supplementary materials), and augmented with label

For REVIEWER #1

1.1 Context

It is a fair point that contextual factors influence the relative support of nudges and regulations. There are numerous prior studies dealing with these effects, and some of them are mentioned in the present study. Nonetheless, the focus of this study was to investigate the role of risk perception and the direct and indirect experience on public support. One must also consider that questions about party preferences and ideologies have been very sensitive in the Hungarian context. COVID-19 issues have been highly politicized, and they are potential hot topics of the incoming elections. Therefore, we decided not to analyze the role of these contextual factors. However, in future research these are absolutely valid directions.

1.2. Method

We included a few sentences about this caveat in the limitations paragraph (end of Conclusion section) because it is a valid concern that not randomising does not correct for any order effect that may have had an effect on the respondents. We argue that this may have affected the results in absolute terms, but it is unlikely to affect the relative differences significantly. As the focus of our research questions was on relative differences between support for nudges and regulation; and support from people with lower or higher levels of perceived risk, not accounting for survey order effect does not weaken our findings.

1.3. 

Nudges in this study were carefully selected in order to assure that people understand the aim and the relevance of these soft policies. Nudges in questions were introduced to the Hungarian society earlier that year, and they became part of people’s everyday life. Hence, we can assume that the rationale behind these nudges were understood by the majority of the Hungrian citizens. Of course, we must admit that there still might have been some people who did not understand the effect of nudges, but other studies revealed that by disclosing the purpose or the underlying mechanisms of nudges, does not change the level of acceptance or the effectiveness of these behavioral policies (Loewenstein et al., 2015; Kroese et al., 2016; Bruns et al., 2018).

2. Edit the document. 

To create a more streamline flow for the readers, the document was thoroughly restructured. We split the Introduction and introduced a Backgrounds section, written a Statistical analysis subsection of the Methods to outline the performed analysis logic, and make the results section more easily interpretable. Combined the Results and Discussion sections to eliminate repetitiveness and close the paper with a partly new Conclusions section focusing on the main policy messages.

3. 

Very fair point that more information about the sample composition would be prudent to disclose. For this reason we included a new table detailing information about the sample, bucketed by the three criteria we list for representative sampling. Also included in the main text the caveat of the sample being truly representative of the Hungarian population: namely the lack of participants above 64 (hence the younger average age the reviewer noted), and the CAWI nature of the survey likely introducing a bias.

As for the reviewers comment on the distribution between the sexes, our sample is representative of the Hungarian population between 18-64 according to the data of Central Statistics Office (KSH) of Hungary.

For REVIEWER #2

1.

We included a short notes to reference analytical results in the abstract as well. 

2. 

We introduced Backgrounds section. A new subsection of Backgrounds (2.5) and a new figure S1 provides more context on the Hungarian development of COVID-19

3. 

We revised the section (2.2) detailing nudges used during the pandemic, introducing the distinction of which studies support their effectiveness and which studies do not. As a result removed one reference that had no empirical results (Park 2020)

From the five advised articles, we also included three but, Huyn (2020), was not referenced in this section because it also does not report about empirical findings. We did not refer to Ding et al. (2020) because their use of word ‘nudge’ is not consistent with the “thalerian” definition we rely on - the lockdown is a much more intrusive phenomenon, taking away, and completely changing possibilities.

4. 

We have written statistical analysis subsection (3.3)

5. 

Removed repetitiveness by combining the two sections, Results and Discussion.

6. 

We included a Conclusion section focusing on the key policy messages

Attachment Submitted filename: Response to Reviewers.pdf Click here for additional data file.

# Peer review: Decision Letter 1

**DOI:** 10.1371/journal.pone.0256241.r003

**Authors:** Valerio Capraro

20 Jul 2021

PONE-D-21-12439R1

Nudging in the time of coronavirus? Comparing public support for soft and hard preventive measures, highlighting the role of risk perception and experience

PLOS ONE

Dear Dr. Dudás,

Thank you for submitting your manuscript to PLOS ONE. After careful consideration, we feel that it has merit but does not fully meet PLOS ONE’s publication criteria as it currently stands. Therefore, we invite you to submit a revised version of the manuscript that addresses the points raised during the review process.

Please submit your revised manuscript by Sep 03 2021 11:59PM. If you will need more time than this to complete your revisions, please reply to this message or contact the journal office at plosone@plos.org. When you're ready to submit your revision, log on to https://www.editorialmanager.com/pone/ and select the 'Submissions Needing Revision' folder to locate your manuscript file.

Please include the following items when submitting your revised manuscript:

- A rebuttal letter that responds to each point raised by the academic editor and reviewer(s). You should upload this letter as a separate file labeled 'Response to Reviewers'.
- A marked-up copy of your manuscript that highlights changes made to the original version. You should upload this as a separate file labeled 'Revised Manuscript with Track Changes'.
- An unmarked version of your revised paper without tracked changes. You should upload this as a separate file labeled 'Manuscript'.

If you would like to make changes to your financial disclosure, please include your updated statement in your cover letter. Guidelines for resubmitting your figure files are available below the reviewer comments at the end of this letter.

If applicable, we recommend that you deposit your laboratory protocols in protocols.io to enhance the reproducibility of your results. Protocols.io assigns your protocol its own identifier (DOI) so that it can be cited independently in the future. For instructions see: http://journals.plos.org/plosone/s/submission-guidelines#loc-laboratory-protocols. Additionally, PLOS ONE offers an option for publishing peer-reviewed Lab Protocol articles, which describe protocols hosted on protocols.io. Read more information on sharing protocols at https://plos.org/protocols?utm_medium=editorial-email&utm_source=authorletters&utm_campaign=protocols.

We look forward to receiving your revised manuscript.

Kind regards,

Valerio Capraro

Academic Editor

PLOS ONE

Journal Requirements:

Please review your reference list to ensure that it is complete and correct. If you have cited papers that have been retracted, please include the rationale for doing so in the manuscript text, or remove these references and replace them with relevant current references. Any changes to the reference list should be mentioned in the rebuttal letter that accompanies your revised manuscript. If you need to cite a retracted article, indicate the article’s retracted status in the References list and also include a citation and full reference for the retraction notice.

Additional Editor Comments (if provided):

One of the reviewers suggest some additional minor improvements. Moreover, I have noticed that you ignored my previous comments on the paper, which you can find in the previous decision letter. Therefore, I am inviting to revise your work again for Plos One. I am looking forward for the revision.

[Note: HTML markup is below. Please do not edit.]

Reviewers' comments:

Reviewer's Responses to Questions

**Comments to the Author**

1. If the authors have adequately addressed your comments raised in a previous round of review and you feel that this manuscript is now acceptable for publication, you may indicate that here to bypass the “Comments to the Author” section, enter your conflict of interest statement in the “Confidential to Editor” section, and submit your "Accept" recommendation.

Reviewer #2: All comments have been addressed

**********

2. Is the manuscript technically sound, and do the data support the conclusions?

The manuscript must describe a technically sound piece of scientific research with data that supports the conclusions. Experiments must have been conducted rigorously, with appropriate controls, replication, and sample sizes. The conclusions must be drawn appropriately based on the data presented. 

Reviewer #2: Yes

**********

3. Has the statistical analysis been performed appropriately and rigorously? 

Reviewer #2: Yes

**********

4. Have the authors made all data underlying the findings in their manuscript fully available?

The PLOS Data policy requires authors to make all data underlying the findings described in their manuscript fully available without restriction, with rare exception (please refer to the Data Availability Statement in the manuscript PDF file). The data should be provided as part of the manuscript or its supporting information, or deposited to a public repository. For example, in addition to summary statistics, the data points behind means, medians and variance measures should be available. If there are restrictions on publicly sharing data—e.g. participant privacy or use of data from a third party—those must be specified.

Reviewer #2: Yes

**********

5. Is the manuscript presented in an intelligible fashion and written in standard English?

PLOS ONE does not copyedit accepted manuscripts, so the language in submitted articles must be clear, correct, and unambiguous. Any typographical or grammatical errors should be corrected at revision, so please note any specific errors here.

Reviewer #2: Yes

**********

6. Review Comments to the Author

Please use the space provided to explain your answers to the questions above. You may also include additional comments for the author, including concerns about dual publication, research ethics, or publication ethics. (Please upload your review as an attachment if it exceeds 20,000 characters)

Reviewer #2: Thank you for the revision, the paper looks much better than the previous version. I am giving another minor revision as I find the language of the Background section to be very condescending. While it is a good thing that the authors have added new references and restructured it well, but please change the tonality of how you are drawing inferences from those cited references. The current writing style of section 2.1 to 2.3 should maintain neutrality to respect the papers that you have cited.

I strongly feel the authors would include the supplementary figures S1 and S2 in the background section as they are very important and should not be thrown as in the appendix.

Please improve the writing of the conclusion section as well, you are starting sentences with 'Of course!....'. Such minor elements of written communication should be improved to match the quality and readership of this journal.

**********

7. PLOS authors have the option to publish the peer review history of their article (what does this mean?). If published, this will include your full peer review and any attached files.

If you choose “no”, your identity will remain anonymous but your review may still be made public.

**Do you want your identity to be public for this peer review?** For information about this choice, including consent withdrawal, please see our Privacy Policy.

Reviewer #2: No

[NOTE: If reviewer comments were submitted as an attachment file, they will be attached to this email and accessible via the submission site. Please log into your account, locate the manuscript record, and check for the action link "View Attachments". If this link does not appear, there are no attachment files.]

While revising your submission, please upload your figure files to the Preflight Analysis and Conversion Engine (PACE) digital diagnostic tool, https://pacev2.apexcovantage.com/. PACE helps ensure that figures meet PLOS requirements. To use PACE, you must first register as a user. Registration is free. Then, login and navigate to the UPLOAD tab, where you will find detailed instructions on how to use the tool. If you encounter any issues or have any questions when using PACE, please email PLOS at figures@plos.org. Please note that Supporting Information files do not need this step.

# Author comment: Author response to Decision Letter 1

**DOI:** 10.1371/journal.pone.0256241.r004

30 Jul 2021

Response to Reviewers

PONE-D-21-12439R1

2nd revision - invite for minor revision

Response to Reviewer #2

a) writing style of section 2.1 to 2.3 should maintain neutrality to respect the papers that you have cited.

Author response: thank you for noticing, we agree the tonality should maintain neutrality and for this reason we rephrased some of our remarks on the cited articles

b) figures should be included in the main text, not relegated to appendix

Author response: we omitted the figures from the main text as per PLOS ONE submission guidelines. We agree they should be included in the main text in the published form of the paper, and noted their place in the manuscript for reference to reviewers and the publication production office.

https://journals.plos.org/plosone/s/submission-guidelines requires: 

 “Do not include figures in the main manuscript file. Each figure must be prepared and submitted as an individual file.”

c) improve the writing of the conclusion section

Author response: the sentences in the conclusion section were adjusted to improve the writing.

Response to Editor

a) the literature review is quite incomplete. I think you should make an effort to review the literature in the best possible ways.

Author response: the literature review was extended and the three mentioned articles were included in section 2.2

Attachment Submitted filename: Response to Reviewers - plos one v2.pdf Click here for additional data file.

# Editor report: Decision Letter 2

**DOI:** 10.1371/journal.pone.0256241.r005

**Authors:** Valerio Capraro

3 Aug 2021

Nudging in the time of coronavirus? Comparing public support for soft and hard preventive measures, highlighting the role of risk perception and experience

PONE-D-21-12439R2

Dear Dr. Dudás,

We’re pleased to inform you that your manuscript has been judged scientifically suitable for publication and will be formally accepted for publication once it meets all outstanding technical requirements.

Within one week, you’ll receive an e-mail detailing the required amendments. When these have been addressed, you’ll receive a formal acceptance letter and your manuscript will be scheduled for publication.

An invoice for payment will follow shortly after the formal acceptance. To ensure an efficient process, please log into Editorial Manager at http://www.editorialmanager.com/pone/, click the 'Update My Information' link at the top of the page, and double check that your user information is up-to-date. If you have any billing related questions, please contact our Author Billing department directly at authorbilling@plos.org.

If your institution or institutions have a press office, please notify them about your upcoming paper to help maximize its impact. If they’ll be preparing press materials, please inform our press team as soon as possible -- no later than 48 hours after receiving the formal acceptance. Your manuscript will remain under strict press embargo until 2 pm Eastern Time on the date of publication. For more information, please contact onepress@plos.org.

Kind regards,

Valerio Capraro

Academic Editor

PLOS ONE

Additional Editor Comments (optional):

Reviewers' comments:

# Editor report: Acceptance letter

**DOI:** 10.1371/journal.pone.0256241.r006

**Authors:** Valerio Capraro

4 Aug 2021

PONE-D-21-12439R2 

Nudging in the time of coronavirus? Comparing public support for soft and hard preventive measures, highlighting the role of risk perception and experience 

Dear Dr. Dudás:

I'm pleased to inform you that your manuscript has been deemed suitable for publication in PLOS ONE. Congratulations! Your manuscript is now with our production department. 

If your institution or institutions have a press office, please let them know about your upcoming paper now to help maximize its impact. If they'll be preparing press materials, please inform our press team within the next 48 hours. Your manuscript will remain under strict press embargo until 2 pm Eastern Time on the date of publication. For more information please contact onepress@plos.org.

If we can help with anything else, please email us at plosone@plos.org. 

Thank you for submitting your work to PLOS ONE and supporting open access. 

Kind regards, 

PLOS ONE Editorial Office Staff

on behalf of

Dr. Valerio Capraro 

Academic Editor

PLOS ONE
